# High-complexity regions in mammalian genomes are enriched for developmental genes

**DOI:** 10.1093/bioinformatics/bty922

**Published:** 2018-11-05

**Authors:** Anton Pirogov, Peter Pfaffelhuber, Angelika Börsch-Haubold, Bernhard Haubold

**Affiliations:** 1 Lehrstuhl für Informatik, RWTH Aachen University, Max-Planck-Institute for Evolutionary Biology, Plön, Germany; 2 Department of Evolutionary Genetics, Max-Planck-Institute for Evolutionary Biology, Plön, Germany; 3 Department of Mathematical Stochastics, Freiburg University, Germany; 4 24306 Plön, Germany

## Abstract

**Motivation:**

Unique sequence regions are associated with genetic function in vertebrate genomes. However, measuring uniqueness, or absence of long repeats, along a genome is conceptually and computationally difficult. Here we use a variant of the Lempel-Ziv complexity, the match complexity, $C_{m}$, and augment it by deriving its null distribution for random sequences. We then apply $C_{m}$ to the human and mouse genomes to investigate the relationship between sequence complexity and function.

**Results:**

We implemented $C_{m}$ in the program macle and show through simulation that the newly derived null distribution of $C_{m}$ is accurate. This allows us to delineate high-complexity regions in the human and mouse genomes. Using our program macle2go, we find that these regions are twofold enriched for genes. Moreover, the genes contained in these regions are more than 10-fold enriched for developmental functions.

**Availability and implementation:**

Source code for macle and macle2go is available from www.github.com/evolbioinf/macle and www.github.com/evolbioinf/macle2go, respectively; $C_{m}$ browser tracks from guanine.evolbio.mgp.de/complexity.

**Supplementary information:**

Supplementary data are available at *Bioinformatics* online.

## 1 Introduction

Since the 1960s DNA reassociation kinetics have been used to quantify the repetitiveness of DNA. In a pioneering study of the reassociation kinetics of CpG islands, Bird *et al.* (1985) discovered that the 1% of the mouse genome making up such islands was unique in the sense that it had no matches elsewhere in the genome. In subsequent years, CpG islands attracted a huge amount of interest as they are associated with the promoters of housekeeping genes (Bird, 1986; The ENCODE Project Consortium, 2012) and influence chromatin structure (Wachter *et al.*, 2014). In addition, Elango and Yi (2011) found that promoters containing CpG islands longer than 2 kb were enriched for developmental genes. In the present study we directly search for unique regions by delineating intervals where exact matches to other parts of the genome are short. 

Uniqueness and repetitiveness are complementary, and Haubold and Wiehe (2006) proposed an early measure of genome repetitiveness, the $I_{r}$. This was based on the lengths of matches starting at every position in the genome. Regions with similarity elsewhere in the genome were characterized by long matches, unique regions by short matches. In a sliding window analysis they found that some regions in the human genome including the *Hox* clusters were characterized by extremely low $I_{r}$. The *Hox* genes encode transcriptional regulators that specify the anterior/posterior axis in all animals (Raff, 1996). Moreover, in the publication of the first draft of the human genome the *Hox* clusters had been singled out as containing very few transposon insertions compared to the rest of the genome (International Human Genome Sequencing Consortium, 2001). Recent transposon insertions would create long exact matches and hence increase the $I_{r}$.

As a statistic, the $I_{r}$ has two disadvantages: Its distribution is unknown, and its implementation too slow for convenient genomics. When Haubold *et al.* (2009) derived the null distribution of match lengths for a random sequence, where all bases are independently drawn given the GC content, this opened the way for constructing a match-based statistic with known null distribution.

The classical match-based statistic for strings is the Lempel-Ziv complexity (Lempel and Ziv, 1976). It is computed from the decomposition of a string, *S*, into a set of substrings, S[i…j], where S[i…j] is the longest substring that has an exact match to the left of S[i]. The number of such maximal matches divided by the length of *S* is the Lempel-Ziv complexity. In a refinement of this measure, Odenthal-Hesse *et al.* (2016) proposed the match complexity, $C_{m}$, where maximal matches of S[i…j] can occur to the left and the right of S[i]. In contrast to the $I_{r}$ and the Lempel-Ziv complexity, $C_{m}$ has known bounds. Its lower bound is 0 for sequences with the minimum number of two matches and its upper bound is reached in long random sequences, for which the expectation of $C_{m}$ is 1.

Here we derive the null distribution of $C_{m}$, which allows us to delineate unique genomic regions. These are defined as regions where the $C_{m}$ is indistinguishable from that found in random sequences. The computation of $C_{m}$, like that of the $I_{r}$ and the Lempel-Ziv complexity, is based on suffix arrays (Ohlebusch, 2013, p. 59ff). A suffix array is essentially an index to some text, in this case the nucleotide sequence of a genome. A standard method for ensuring programs based on this technology are fast, is to separate index computation, which may take hours, from index querying, which is often a matter of seconds. Our implementation of $C_{m}$, macle for MAtch CompLExity, makes use of this separation leading to querying times for the complete human genome of half a minute or less.

We apply macle to the human and mouse genomes. Since we are particularly interested in regions unique within these genomes, we first need to establish by simulation that the newly derived null distribution of $C_{m}$ is accurate. Next, we scan the human and mouse genomes and ask two questions: First, are highly complex regions enriched for promoters? Second, are the genes with promoters in high-complexity regions enriched for particular functions? We find that high-complexity regions are mildly enriched for promoters, but that these promoters are strongly enriched for developmental genes.

## 2 Methods and data

### 2.1 The match complexity

The match complexity, $C_{m}$, was first described by Odenthal-Hesse *et al.* (2016). Consider a string S=S[1…L] and extend it by S[L+1]=$, where $ is a unique character. Given the first match factor $S[1\ldots F_{1}]$, where $F_{1}=\max\{k:S[1\ldots k]$ matches elsewhere in *S*}, we define recursively the *n*th match factor $S[F_{n-1}+1\ldots F_{n}]$, which ends at $F_{n}=\max\{k:S[F_{n-1}+1\ldots k]$ matches elsewhere in *S*}. We stop with the *N*th match factor if *F_N_* = *L* and set $N_{L}:=N$. For example, S=CGGGCGGGCT has $N_{L}=3$ factors, CGGGC.GGGC.T.

Following Odenthal-Hesse *et al.* (2016), the match decomposition of a string is computed from its sorted suffixes. Table 1 shows the sorted suffixes of *S* as column suf[i]. The suffix array, sa[i], abstracts from this the starting positions. It is ‘enhanced’ by the largest common prefix array, lcp[i], which denotes the length of the longest prefix match between suf[i] and suf[i−1]; lcp[1]=−1, as there is no suffix to compare with (Ohlebusch, 2013, p. 79ff). To decompose *S*, the lcp array is traversed in the order in which the suffixes appear in *S*. The mapping between positions in *S* and in sa is the inverse suffix array, isa[sa[i]]=i. As summarized in Algorithm 1, the longest match starting at position *i* is determined by looking up lcp[isa[i]] and lcp[isa[i]+1]. The greater of these is the length of the desired match factor. The algorithm reports the factor, skips it and repeats until it has traversed the entire sequence.

**Table 1.** Enhanced suffix array of S=CGGGCGGGCT

*i*	sa[i]	lcp[i]	isa[i]	suf[i]
1	1	−1	1	CGGGCGGGCT
2	5	5	8	CGGGCT
3	9	1	6	CT
4	4	0	4	GCGGGCT
5	8	2	2	GCT
6	3	1	9	GGCGGGCT
7	7	3	7	GGCT
8	2	2	5	GGGCGGGCT
9	6	4	3	GGGCT
10	10	0	10	T

**Algorithm 1 Computing the match factor decomposition**

**Require:** *S* {input sequence}

**Require:** lcp {longest common prefix array of *S*}

**Require:** isa {inverse suffix array of *S*}

**Require:** *n* {length of *S*}

**Ensure:** Match decomposition

 1: i←1 {set index to first position in *S*}

 2: lcp[n+1]←0 {prevent out of bounds error}

 3: **while**i≤n**do**

 4: $l_{1}\leftarrow \text{lcp}[\text{isa}[i]]$

 5: $l_{2}\leftarrow \text{lcp}[\text{isa}[i]+1]$

 6: $j\leftarrow i+\max(l_{1},l_{2},1)-1$

 7: reportMatchFactor(S[i…j])

 8: i←j+1

If we apply Algorithm 1 to the enhanced suffix array of *S* in Table 1, we first look up lcp[isa[1]]=lcp[1], which is −1, and lcp[2], which is 5. Hence the first match factor S[1…5] is reported and the algorithm repeats by looking up lcp[isa[6]]=lcp[9]=4 and lcp[10]=0. The second factor S[6…9] is reported, and so on.

Computation of the lcp array is carried out in time proportional to the length of the corresponding sa by first computing its isa (Ohlebusch, 2013, p. 79ff). In practice, suffix array construction consumes the bulk of the resources necessary for match decomposition.

In order to define $C_{m}$, we need the following three quantities for a sequence, *S*, of length *L*: First, $C_{o}=N_{L}/L$ is the observed number of match factors per base; second, $C_{i}=2/L$ is the theoretical minimum; third, $C_{a}$ is the expected match count per base in a random sequence of length *L* with the same GC-content as *S*, which we explain below. With these quantities, we define

$$C_{m}=\frac{C_{o}-C_{i}}{C_{a}-C_{i}}.$$

Subtraction of $C_{i}$ ensures that $C_{m}$ is bounded by 0 and an expectation of 1.

To compute $C_{a}$, we use the distribution of the lengths, $Y_{i}^{*}$, of the longest matches starting at position *i* in a random sequence of GC-content 2*p* (Haubold *et al.*, 2009):

(1) $$P(Y_{i}^{*}<x)\approx \sum_{\underline{k}}\left(\begin{array}{c} x\\ \underline{k} \end{array}\right)p^{\underline{k}}{\left(1-{\underline{p}}^{\underline{k}}\right)}^{L}.$$

Here, $\underline{k}$ is the vector of nucleotide counts, $\underline{k}=(k_{A},k_{C},k_{G},k_{T})$, which sum to the threshold length, $x=k_{A}+k_{C}+k_{G}+k_{T}$; and $\underline{p}$ is the vector of nucleotide frequencies, $\underline{p}=(0.5-p,p,p,0.5-p)$. From Equation (1) we compute the mean, *μ*, and variance, $\sigma^{2}$, of the match length distribution:

$$\begin{array}{c} \mu :=1/C_{a}=E\left[Y_{i}^{*}\right]=\sum_{x=1}^{L}x(P(Y_{i}^{*}<x+1)-P(Y_{i}^{*}<x)),\\ \sigma^{2}:=V\left[Y_{i}^{*}\right]\\ =\sum_{x=1}^{L}x^{2}(P(Y_{i}^{*}<x)-P(Y_{i}^{*}<x-1))-{\left(E\left[Y_{i}^{*}\right]+1\right)}^{2}. \end{array}$$

Given the match decomposition of, say, the human genome, we wish to compute local values of $C_{m}$ by sliding a window of length *W* across the decomposition, and computing $C_{o},\;C_{i}$ and $C_{a}$ with respect to the current window: $C_{o}=N_{W}/W$, $C_{i}=2/W$ and $C_{a}=1/\mu$.

We define highly complex regions as those that are indistinguishable from random. In order to detect such regions, we need to calculate appropriate threshold values, or quantiles, of $C_{m}$. For this purpose we model the null distribution of $C_{m}$ by a normal distribution. This is justified by assuming that L≫W≫1. Now let *N_i_* denote the number of factors up to position *i*. Then ${\left(N_{i}\right)}_{i=0,1,2,\ldots }$ is a renewal process, since its increments are—by assumption—independent and equally distributed according to the distribution of $Y_{i}^{*}$. According to the central limit theorem for renewal processes (Serfozo, 2009, Example 67),

$$N_{W}\approx \frac{W}{\mu }+B_{W},$$

where *B_W_* is normally distributed with mean 0 and variance $W\sigma^{2}/\mu^{3},\;B_{W}∼N(0,\sigma^{2}W/\mu^{3})$. This leads to

(2) $$C_{m}\approx \frac{\mu }{W}N_{W}\approx 1+\frac{\mu }{W}B_{W}∼N\left(1,\frac{\sigma^{2}}{\mu W}\right),$$

which allows us to approximate quantiles for $C_{m}$ using the quantile function

$$F^{-1}(p)=1+\sqrt{2}\frac{\sigma^{2}}{\mu W}{\text{erf}}^{-1}(2p-1),$$

where *p* is the probability covered up to that point, say 5%, and erf is the error function.

### 2.2 Implementation

We used our program macle to compute $C_{m}$ in sliding windows of length 10 kb, which advanced in steps of 1 kb, thus generating sets of overlapping windows. Macle is written in C++ and calls the software library libdivsufsort (available from github) for suffix array computation. This library implements one of the fastest suffix sorting algorithms known, the divSufSoft algorithm recently described by Fischer and Kurpicz (2017). Given the human genome in FASTA format, it is first indexed, and can then be queried repeatedly.

We wrote the program macle2go in Go to annotate the output of macle. Macle2go implements three functions, quantile, annotate and enrichment.

Quantile implements the quantile computation outlined above.

Annotate first identifies the *n* windows of a given minimum $C_{m}$. It then finds the $g_{o}$ genes whose promoters intersect one or more of these *n* windows; we defined the promoter of a gene as the 4 kb interval centered on its transcription start site (Saxonov *et al.*, 2006). Annotate also repeatedly draws *n* random windows to determine the number of genes expected by chance alone, $g_{e}$. In addition, it counts the number of times, *f*, that *n* windows are found containing $\geq g_{o}$ genes in *i* iterations. Then the *P*-value of $H_{0}:g_{o}=g_{e}$ is P=f/i, or P<1/i, if *f *=* *0.

Enrichment connects the genes found by annotate to the functional categories of the gene ontology (GO) (The Gene Ontology Consortium, 2000). The result is a list of GO terms and the number of genes observed in that category. Enrichment also carries out a randomization procedure similar to that used by annotate to test the significance of finding more genes than expected in a particular GO category.

### 2.3 Data

Human genome version GRCh38.p2 and mouse genome version GRCm38.p3 were used throughout. The RefGene annotation data for both organisms was downloaded from the UCSC genome browser. To connect genes to GO-terms, we used the files Homo_sapiens.gene_info, Mus_musculus.gene_info and gene2go from the NCBI website. In addition, we downloaded CpG islands from the UCSC genome browser. All the primary data files mentioned here are posted together with our $C_{m}$ browser tracks for human and mouse.

## 3 Results

### 3.1 Null distribution

**Fig. 1.**
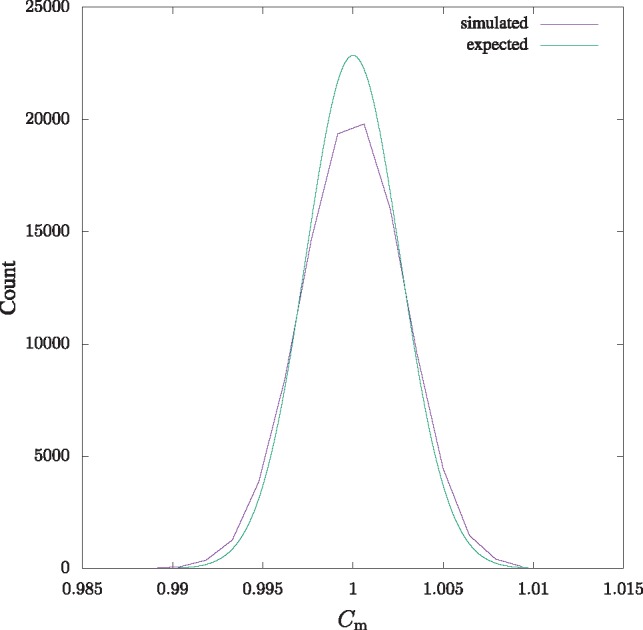
The simulated and expected null distribution of $C_{m}$. The simulated distribution was computed from a 100 Mb random sequence with a 10 kb sliding window. The expected distribution is given in Equation (2)

To check the accuracy of the null distribution in Equation (2), we simulated a random 100 Mb sequence and carried out a sliding window analysis with 10 kb windows. Figure 1 shows the distribution of local $C_{m}$ values compared to Equation (2). The fit is not perfect, but reasonable.

### 3.2 Time and memory consumption

**Fig. 2.**
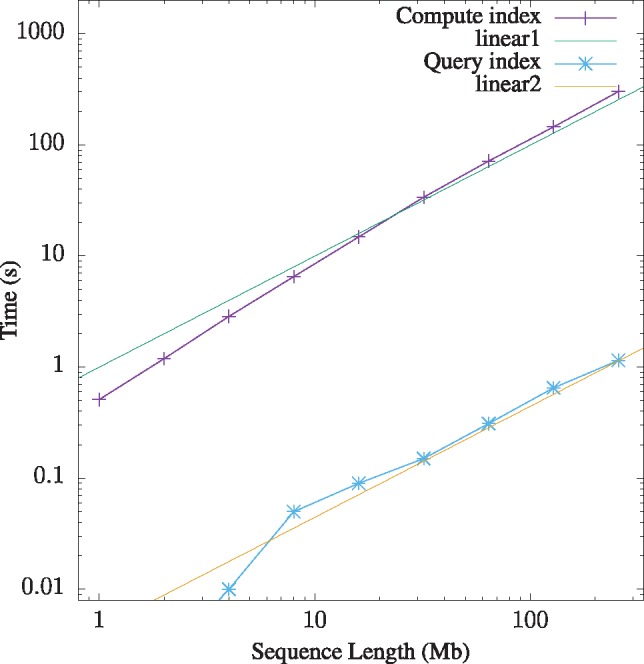
Run time of macle as a function of sequence length. Measurements made from single random sequences

We investigated the resource consumption of macle using simulated sequences. Our test computer ran Ubuntu 18.04 on Intel Xeon 2.10 GHz processors with 256 GB RAM. Macle first computes a permanent index, which can then be queried repeatedly. Figure 2 shows that index construction is slightly more than linear in the length of the input sequence. Still, 1 Mb per s can be taken as a rule of thumb. Index traversal, on the other hand, is expected to take time proportional to index length, which in turn is proportional to sequence length. Figure 2 shows that querying the index by sliding a window across the entire input sequence is indeed linear in sequence length and takes 1.15 s for 256 Mb, while the corresponding index construction takes 302.3 s, that is, over 250 times longer.

Memory consumption of index construction and querying is strictly linear in the length of the input sequence (not shown). Construction consumes approximately 35 MB per Mb, querying four times less, 8.4 MB per Mb.

### 3.3 Application to the human and mouse genomes

**Fig. 3.**
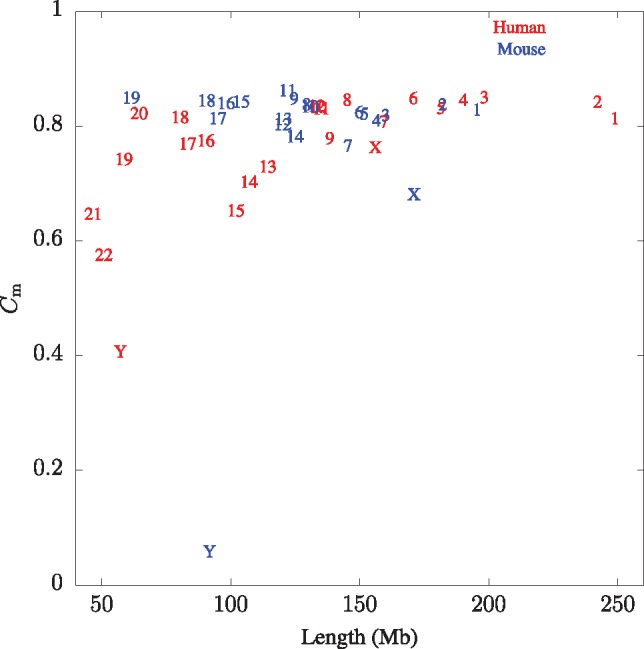
Match complexity of human and mouse chromosomes as a function of their length. Some labels are superimposed, especially human chromosomes 10, 11 and 12, and mouse chromosomes 8 and 10

Indexing the 3.1 Gb of the human genome took 1 h, 19 min, 3 s and 128.2 GB RAM. Similarly, indexing the 2.7 Gb of the mouse genome took 1 h, 8 min, 33 s and 111.1 GB RAM. The first thing we calculated off these indexes was genome-wide $C_{m}$, which is 0.8071 in human and 0.7868 in mouse. In other words, the mouse genome is overall slightly less complex, or more repetitive, than the human genome. However, these genome-wide values hide a large diversity of chromosome-specific complexity. Figure 3 shows the $C_{m}$ for the 19 mouse autosomes, the 22 human autosomes and their sex chromosomes. In humans the chromosome-wide complexity varies between 0.40 in the Y chromosome and 0.85 in chromosome 3. Interestingly, shorter chromosomes have significantly lower complexity than longer chromosomes with a correlation of *r *=* *0.61 ($P=1.5\times 10^{-3}$). In mouse, $C_{m}$ ranges from an extraordinarily low $C_{m}=0.06$ in the Y chromosome to $C_{m}=0.86$ in chromosome 11. In contrast to human, there is no correlation between chromosome length and complexity (*r *=* *0.17, *P *=* *0.45). Notice also that in mouse the X chromosome ($C_{m}=0.68$) has the second lowest complexity, while the human X chromosome ($C_{m}=0.76$) has a complexity similar to that of equally long autosomes.

**Fig. 4.**
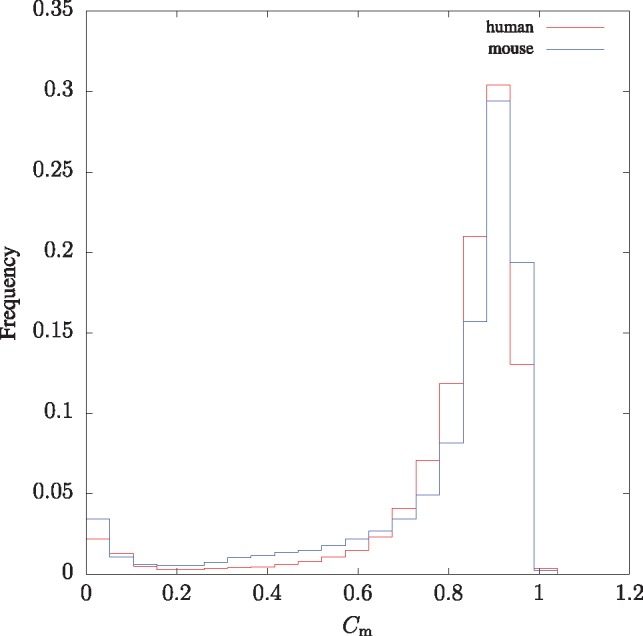
Histogram of $C_{m}$ values across the human and mouse genomes for 10 kb sliding windows

We now zoom into the genomes by carrying out sliding window analyses. Figure 4 shows the frequency distribution of $C_{m}$ in 10 kb sliding windows across the human and mouse genomes. This is bimodal with a large mode at 0.91 representing the bulk of both genomes, and a smaller mode at 0.05 for highly repetitive regions. Notice that mouse has a larger proportion of low-complexity regions than human, presumably due to the extremely low complexity of its Y chromosome (Fig. 3).

**Fig. 5.**
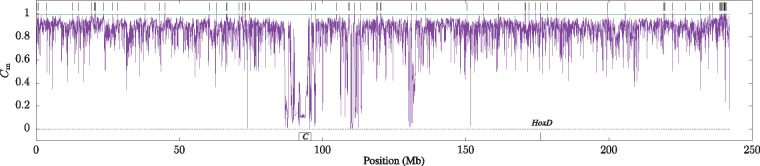
Match complexity, $C_{m}$, in 10 kb sliding windows along human chromosome 2. The green horizontal line marks the complexity threshold [Equation (2)] and the vertical bars the regions with complexity greater or equal to that threshold. *C*, centromere; *HoxD*, location of the *HoxD* gene cluster (Color version of this figure is available at *Bioinformatics* online.)

Next, we investigate how the $C_{m}$ values summarized in Figure 4 are distributed along individual chromosomes. Figure 5 shows $C_{m}$ along human chromosome 2, which contains one of the four human *Hox* clusters, *HoxD*, at 176.2 Mb. The green horizontal line at $C_{m}=0.9954$ is the 5% quantile of the $C_{m}$ distribution in random sequences obtained from Equation (2) and delineates high-complexity regions. There are 79 such regions ranging from 10 kb to 77 kb and totaling 1.2 Mb, or 0.50% of chromosome 2. Figure 5 depicts these regions as vertical lines. At the other extreme of the $C_{m}$ distribution is the centromere, which is characterized by 4 Mb of very low $C_{m}$.

**Fig. 6.**
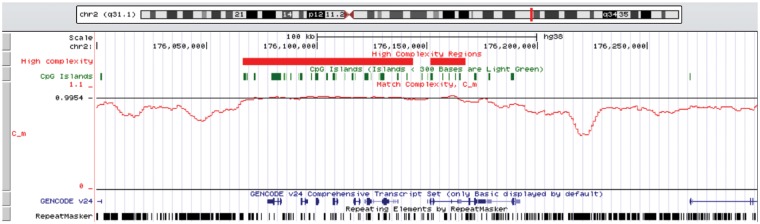
High-complexity regions and $C_{m}$ in sliding windows (red), CpG islands (green), condensed genes (blue) and RepeatMasker annotations around the human *HoxD* cluster, chr2: 176 000 000–176 300 000, rendered in the UCSC genome browser (Color version of this figure is available at *Bioinformatics* online.)

In order to visualize how $C_{m}$ highlights genes, Figure 6 shows our $C_{m}$ results integrated with the UCSC genome browser in the *HoxD* region. Notice the two 100 kb-spanning regions of high complexity. These overlap a large portion of the *HoxD* genes shown as the ‘GENCODE’ track. They also correspond to a high density of CpG islands and a low density of RepeatMasker elements.

In total, the human genome contains 1234 high-complexity intervals constructed by merging the overlapping elements among 5867 high $C_{m}$ windows (Supplementary Table S1). They range in length from 10 kb to 77 kb, totaling 17.3 Mb or 0.56% of the genome. The three longest regions, chr2: 176 066 579–176 143 578, chr7: 27 135 698–27 208 697 and chr12: 53 948 018–54 010 017 are centered on *HoxD*, *HoxA* and *HoxC*, respectively. The remaining *Hox* cluster, *HoxB*, is covered by two intervals, chr17: 48 573 129–48 608 128 and chr17: 48 609 129–48 634 128. The 1234 intervals contain 1443 genes, while the expected number of genes based on repeatedly drawing 5867 random windows is 876.55. This 1.65-fold gene enrichment is highly significant ($P<10^{-4}$).

In mouse there are 2908 high-complexity windows that merge into 772 distinct intervals (Supplementary Table S2). These range in length from 10 to 56 kb totaling 10.1 Mb or 0.37% of the mouse genome. We were surprised to find that the 56 kb interval chr2: 76 703 660–76 759 659 contains no promoter. It does, however, intersect the gene encoding titin, *Ttn*, a component of muscles (chr2: 76 703 983–76 982 547). Homozygous mutations in *Ttn* lead to developmental defects and premature death (www.informatics.jacx.org). In total, the promoters of 958 genes are found in these intervals, compared to an expectation of 401.94. This amounts to a 2.38-fold enrichment of genes, which include members of *HoxA*, *HoxB* and *HoxD*; *HoxC* is missing, as its $C_{m}$ remains slightly below the cutoff.

### 3.4 Functional enrichment

The 1443 human promoters in high-complexity regions cluster in 211 biological processes with at least 10 members. We ran our Monte-Carlo procedure to test whether the observed number of genes in a particular GO category is larger than expected by chance alone with 10^8^ iterations. This resulted in 45 categories enriched with maximal significance ($P<10^{-8}$). When Bonferroni-corrected for the 211 tests, this amounts to $P<2.1\times 10^{-6}$. The degree of enrichment ranged from 18.7 to 2.1 (Supplementary Table S3). The enriched categories are involved in cell differentiation, morphogenesis and organ development. Table 2 lists the top 10 enriched categories. The genes underlying these functional categories contain many well-known transcription factors, including *Lhx* in the category ‘spinal cord association neuron differentiation’, *Fox* in ‘dopaminergic neuron differentiation’ and *Hox* in ‘anterior/posterior pattern specification’ (Supplementary Table S3).

**Table 2.** Functional enrichment in human high-complexity regions, $P<2.1\times 10^{-6}$

#	Process	*C*	*O*	*E*	*O* / *E*
1	Spinal cord association neuron diff.	13	11	0.6	18.7
2	Dopaminergic neuron diff.	21	14	1.0	14.0
3	Neuron fate spec.	15	11	0.8	13.8
4	Cell fate determination	17	12	0.9	13.4
5	Middle ear morph.	20	10	1.0	10.5
6	Anterior/posterior pattern spec.	80	41	4.0	10.2
7	Embryonic skeletal system morph.	38	20	2.0	10.1
8	Thyroid gland dev.	24	13	1.3	10.1
9	…branching…in ureteric bud morph.	19	10	1.0	10.1
10	Branching…in ureteric bud morph.	43	21	2.2	9.7

*C*, count of genes in genome; *O*, observed number of genes; *E*, expected number of genes; diff., differentiation; spec., specification; morph., morphogenesis; dev., development.

The 958 mouse genes in high-complexity regions cluster in 173 processes with at least 10 members, of which 51 are maximally significant ($P<10^{-8}\times 173=1.7\times 10^{-6}$) with enrichment factors ranging from 14.4 to 2.8 (Supplementary Table S4). Again, they are involved in a broad range of developmental processes. Table 3 lists the top 10 enriched categories. As in the case of human, the genes underlying these functional categories contain numerous widely studied transcription factors, such as *Pax* in ‘cell fate determination’, *Gata* in ‘tissue development’ and *Hox* in ‘embryonic skeletal system morphogenesis’.

**Table 3.** Functional enrichment in mouse high-complexity regions, $P<1.7\times 10^{-6}$

#	Process	*C*	*O*	*E*	*O* / *E*
1	Cell fate determination	18	10	0.7	14.4
2	Dopaminergic neuron diff.	29	13	1.1	11.8
3	Tissue dev.	27	11	1.0	11.3
4	Anatomical structure formation…	27	12	1.1	11.3
5	Ventricular septum morph.	38	16	1.4	11.1
6	Branching…in blood vessel morph.	40	16	1.5	10.8
7	Embryonic forelimb morph.	36	15	1.4	10.5
8	Embryonic skeletal system dev.	43	17	1.7	10.2
9	Embryonic skeletal system morph.	56	20	2.0	9.9
10	Metanephros dev.	37	13	1.3	9.8

*C*, count of genes in genome; *O*, observed number of genes; *E*, expected number of genes; diff., differentiation; morph., morphogenesis; dev., development.

Elango and Yi (2011) reported that CpG islands longer than 2 kb are also associated with developmental genes. So we asked, whether our high-complexity regions coincided with CpG islands in general, and specifically with long CpG islands. In human, 88% of high-complexity regions intersect one or more of the 30 477 CpG islands. In mouse almost the same proportion, 87%, of high-complexity regions intersect one or more of its 16 023 CpG islands. This proportion drops if we restrict the analysis to long CpG islands, of which the human genome contains 1426. Only 35% of high-complexity regions intersect a member of this class of CpG islands. Similarly, in mouse only 19% of high-complexity regions intersect a long CpG island.

## 4 Discussion

The relationship between raw nucleotide sequence and its biological function has been at the center of molecular biology since the discovery of the double helix. An early insight was that the genomes of eukaryotes are riddled with non-functional sequences, especially transposons. Devising fast methods for finding repetitive elements has been a major concern of bioinformatics, as genomes are routinely delivered with repeats annotated (Fig. 6) or masked (Bao *et al.*, 2015). In human, approximately half the genome is masked.

Instead of identifying repeats, we have concentrated on finding repeat-free regions, because uniqueness as defined by reassociation kinetics has been linked to CpG islands for decades (Bird *et al.*, 1985), and CpG islands are functional markers in vertebrate genomes. We measure uniqueness using the match complexity, $C_{m}$ (Odenthal-Hesse *et al.*, 2016), thereby effectively carrying out an *in silico* reassociation experiment. $C_{m}$ is calculated by augmenting suffix array techniques (Algorithm 1) with the mathematics of the match length distribution summarized in Equation (2). This equation is based on the assumption that the number of factors in a long window is approximately normally distributed, which fits the simulations (Fig. 1). Equipped with this formalism we computed $C_{m}$ across the human and mouse genomes, and connected the results with genes and their functions.

Our program macle is designed for efficiency. The enhanced suffix array it computes is written to disk in binary form to allow querying of arbitrary regions hundreds of times more quickly than the one-off index construction (Fig. 2). However, further speedup of index computation might be forthcoming due to the recent publication of a parallel version of the divSufSort algorithm on which macle is based (Labeit *et al.*, 2016). In contrast, the hypothesis testing in macle2go already runs in parallel, as the problem of repeatedly drawing sets of windows easily lends itself to this type of optimization.

When querying individual chromosomes, the $C_{m}$ values for human in Figure 3 are more widely scattered than for mouse. The one exception to this rule is the mouse Y chromosome, which is a true outlier among the chromosomes studied with $C_{m}=0.06$. Correspondingly, the sliding window graph of this chromosome contains long stretches of low $C_{m}$ and looks different from all other chromosomes (see online browser tracks). This might come as a surprise since the male-specific region of the Y chromosome is 99.9% euchromatic and contains approximately 700 protein-coding genes (Soh *et al.*, 2014). However, these genes form an ‘ampliconic’ structure consisting of recently duplicated copies of genes involved in spermatogenesis.

The mouse Y chromosome illustrates a peculiarity of the $C_{m}$: Regions with low complexity are usually assumed to be gene-poor and heterhochromatic. The mouse Y chromosome shows that this need not be the case. A low $C_{m}$ merely indicates a recent duplication, regardless of the length of the region involved, or its copy number.

In contrast, high $C_{m}$, the focus of this study, has an unambiguous interpretation: It indicates the absence of recent duplication, perhaps due to selection against it. Approximately 0.50% of chromosome 2 is high-complexity (Fig. 5), which is close to the 0.56% high-complexity across the entire human genome. Haubold and Wiehe (2006) had previously observed in a less systematic fashion that such regions contained developmental genes such as members of the four *Hox* clusters.

We carried out a comprehensive sliding window analysis to study this rigorously. Its most basic parameter is window length, which we arbitrarily set to 10 kb, as the enrichment for developmental genes remains highly significant in mouse and human regardless of whether windows of 5, 10 or 20 kb are analyzed: In humans the most highly enriched categories for 5 and 20 kb windows are ‘spinal cord association neuron differentiation’, and ‘proximal/distal pattern formation’, respectively (Supplementary Tables S5 and S6), which fits with the top category for 10 kb windows, which like for 5 kb windows is ‘spinal cord association neuron differentiation’ (Table 2). Similarly, in mouse the most highly enriched category detected with 5 kb windows is ‘embryonic skeletal system development’, and with 20 kb windows ‘anterior/posterior pattern specification’ (Supplementary Tables S7 and S8). These developmental categories fit the category most highly enriched using 10 kb windows, ‘cell fate determination’ (Table 3).

However, with increasing window length the high-complexity fraction of the genome decreases. In human, 5 kb windows cover 63.8 Mb, 10 kb windows 17.3 Mb and 20 kb windows cover merely 4.7 Mb (for raw data see Supplementary Tables S1, S9 and S10). Similarly, in mouse, 5 kb windows cover 47.8 Mb, 10 kb windows 10.1 Mb and 20 kb windows cover merely 1.0 Mb (Supplementary Tables S2, S11 and S12). So the numerical details of our analysis depend strongly on the window size, but not the general conclusion that high-complexity regions in human and mouse are enriched for developmental genes.

Another potential issue with our analysis is our decision to count promoters intersecting the high-complexity regions rather than whole genes. However, we have programmed our annotation tool, macle2go, such that it can also use whole genes as the unit of comparison. Again, the choice makes no qualitative difference (not shown).

Finally, we investigated the relationship between high-complexity regions and CpG islands. Over 85% of high-complexity regions in human and mouse contain CpG islands. The preponderance of high-complexity regions in GC-rich regions is perhaps not surprising, because fewer matches are found in regions where the local GC content is significantly higher than the global GC content, as is the case in CpG islands. ‘General’ CpG islands are not enriched in developmental genes, while CpG islands longer than 2 kb are (Elango and Yi, 2011). However, only between one fifth and one third of our high-complexity regions intersect long CpG islands, and the high-complexity region in mouse *Ttn* contains neither short nor long CpG islands. Still, we suspect that both attributes, high-complexity and CpG enrichment, are tied to the same phenomenon, biological function; the difference being that high match complexity captures a particular subset of functions, those sensitive to transposon insertion and copy number variation.

We conclude that the match complexity can be used to identify genomic regions highly enriched in developmental genes. The type of analysis established in this study is applicable to any genome with complete sequence and reasonably comprehensive annotation. We therefore plan to analyze the high-complexity regions in other mammals and then across the vertebrates. Genomes with less complete annotations than human or mouse are likely to result in more regions lacking annotation. Among these, those with the highest complexity would be the most promising candidates for further, functional study.

*Conflict of Interest*: none declared.

## Supplementary Material

bty922_Supplementary_Tables
