# High-complexity regions in mammalian genomes are enriched for developmental genes

**DOI:** 10.1093/bioinformatics/btab639

**Published:** 2021-09-16

**Authors:** Anton Pirogov, Peter Pfaffelhuber, Angelika Börsch-Haubold, Bernhard Haubold

*Bioinformatics* (2019) doi:http://dx.doi.org/10.1093/bioinformatics/bty922

In the originally published version of this manuscript, there was an error in the ‘Availability and implementation’ section of the Abstract.

The URL for the browser tracks should be: guanine.evolbio.mpg.de/complexity

This detail has been corrected only in this corrigendum to preserve the published version of record.

